# {2-[(5-Bromo-2-oxidobenzyl­idene)amino-κ^2^ *N*,*O*]-3-methyl­penta­noato-κ*O*}(1,10-phenanthroline-κ^2^ *N*,*N*′)copper(II) dihydrate

**DOI:** 10.1107/S1600536808009495

**Published:** 2008-05-03

**Authors:** Zheng Liu, Yong-Liao Wang, Yuan Wang

**Affiliations:** a Key Laboratory of Non-ferrous Metal Materials and Processing Technology, Department of Materials and Chemical Engineering, Guilin University of Technology, Ministry of Education, Guilin 541004, People’s Republic of China

## Abstract

In the title compound, [Cu(C_13_H_14_BrNO_3_)(C_12_H_8_N_2_)]·2H_2_O, the Cu^II^ atom is penta­coordinated in a square-pyramidal geometry. The crystal packing is stabilized by O—H⋯O hydrogen bonds.

## Related literature

For related literature, see: Feng *et al.* (2007 ▶); Li *et al.* (2006 ▶); Royles & Sherrington (2000 ▶); Jiang *et al.* (2003 ▶); Kettmann *et al.* (1993 ▶); Zhang (2006 ▶); Zhang *et al.* (2003 ▶).
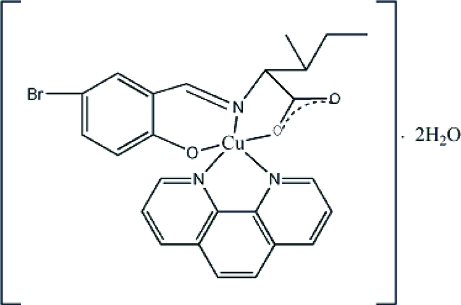


## Experimental

### 

#### Crystal data

[Cu(C_13_H_14_BrNO_3_)(C_12_H_8_N_2_)]·2H_2_O
*M* *_r_* = 591.94
Monoclinic, 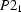

*a* = 10.6184 (18) Å
*b* = 6.0520 (16) Å
*c* = 19.777 (3) Å
β = 93.481 (2)°
*V* = 1268.5 (4) Å^3^
*Z* = 2
Mo *K*α radiation
μ = 2.48 mm^−1^
*T* = 298 (2) K
0.65 × 0.10 × 0.07 mm

#### Data collection

Bruker SMART CCD area-detector diffractometer
Absorption correction: multi-scan (*SADABS*; Sheldrick, 1996 ▶) *T*_min_ = 0.296, *T*_max_ = 0.846
6692 measured reflections
4255 independent reflections
2269 reflections with *I* > 2σ(*I*)
*R*_int_ = 0.053

#### Refinement

*R*[*F* ^2^ > 2σ(*F* ^2^)] = 0.054
*wR*(*F* ^2^) = 0.074
*S* = 0.96
4255 reflections
318 parameters
1 restraint
H-atom parameters constrained
Δρ_max_ = 0.52 e Å^−3^
Δρ_min_ = −0.26 e Å^−3^
Absolute structure: Flack (1983 ▶), 1785 Friedel pairs
Flack parameter: 0.054 (14)

### 

Data collection: *SMART* (Bruker, 2004 ▶); cell refinement: *SAINT* (Bruker, 2004 ▶); data reduction: *SAINT* and *SHELXTL* (Sheldrick, 2008 ▶); program(s) used to solve structure: *SHELXS97* (Sheldrick, 2008 ▶); program(s) used to refine structure: *SHELXL97* (Sheldrick, 2008 ▶); molecular graphics: *SHELXTL*; software used to prepare material for publication: *SHELXTL*.

## Supplementary Material

Crystal structure: contains datablocks global, I. DOI: 10.1107/S1600536808009495/bt2691sup1.cif

Structure factors: contains datablocks I. DOI: 10.1107/S1600536808009495/bt2691Isup2.hkl

Additional supplementary materials:  crystallographic information; 3D view; checkCIF report

## Figures and Tables

**Table 1:** Hydrogen-bond geometry (Å, °)

*D*—H⋯*A*	*D*—H	H⋯*A*	*D*⋯*A*	*D*—H⋯*A*
O5—H28⋯O1	0.85	2.06	2.904 (8)	174
O5—H29⋯O4	0.85	1.87	2.708 (10)	168
O4—H26⋯O5^i^	0.85	2.06	2.853 (8)	156
O4—H27⋯O2^ii^	0.85	2.02	2.746 (7)	143

Symmetry codes: (i) 
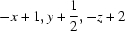
; (ii) 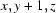.

**Fig. 1.**
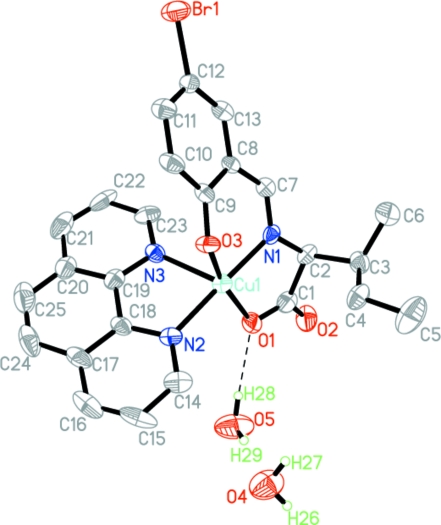
A view of the title compound, showing 30% probability displacement ellipsoids.

**Fig. 2.**
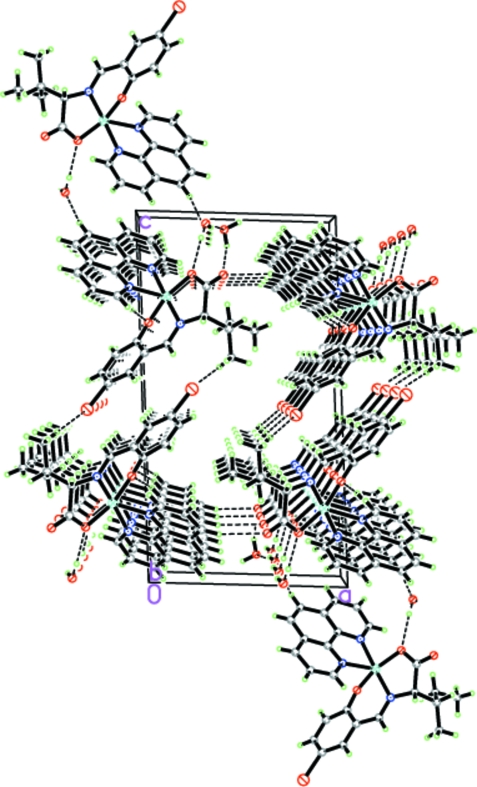
Packing diagram of the title compound.

## supplementary crystallographic information

### Comment

Schiff base complexes play an important role in   antibacterial and catalytic performance, and have attracted widespread interest by researchers (Jiang *et al.*, 2003; Kettmann  *et al.*, 1993; Zhang, 2006). Meanwhile, Schiff base complexes containing isoleucine have been studied because they are of great significance in the biological and medical field (Royles *et al.*, 2000;   Feng *et al.*, 2007; Li *et al.*, 2006).

The central Cu^II^ atom is penta-coordinated (Fig.1). The quadratic planar is composed by O1, N2, O3and N1. The Schiff base forms two chelating rings(O1—C1—C2—N1—Cu1 and N1—C7—C8—C9—O3- Cu1) to the Cu^II^atom, with a diheral angle of 19.6 (4)° which is in the range observed for many copper Schiff base complexes. The N3 atom occupies the axial position  with a N—Cu length of 2.229 (6) Å, comparing with the equatorial Cu—N bond lengths [Cu1—N1 1.924 (6)Å and N2—Cu1 1.975 (6)Å]. The crystal packing is stabilized by  O—H···O hydrogen bonds (Fig. 2).

### Experimental

5-Bromo-2-hydroxy-benzaldehyde(0.5 mmol, 100.5 mg) was dissolved in hot ethanol(5 ml), then a mixture of D,*L*-isoleucine (0.5 mmol, 65.6 mg) and sodium hydroxide  (1.0 mmol, 40 mg) was added. After stirring for 1 h, the copper dinitrate trihydrate(0.5 mmol, 120.8 mg) was added and refluxed for another 2 h. At last, an ethanol solution of Phen(0.5 mmol, 99.1 mg) was dropped gradually into to the reaction mixture and refluxed for further 3 h (Zhang *et al.*, 2003;   Zhang *et al.*, 2006). The obtained green solution was filtered and held at room temperature for ten days, whereupon green crystals suitable for X-ray diffraction were obtained (yield: 45.2%, based on Cu).

### Refinement

All H atoms were positioned geometrically and were treated as riding atoms with C–H distances of 0.93 Å and *U*_iso_(H) = 1.2 *U*_eq_(C) and with O–H distances of 0.85 Å and *U*_iso_(H) = 1.5 *U*_eq_(O). The methyl groups were allowed to rotate but not to tip.

### Crystal data

**Table:**

[Cu(C_13_H_14_BrNO_3_)(C_12_H_8_N_2_)]·2H_2_O	*F*_000_ = 602
*M**_r_* = 591.94	*D*_x_ = 1.550 Mg m^−^^3^
Monoclinic, *P*2_1_	Mo *K*α radiation λ = 0.71073 Å
*a* = 10.6184 (18) Å	Cell parameters from 1263 reflections
*b* = 6.0520 (16) Å	θ = 2.2–18.0º
*c* = 19.777 (3) Å	µ = 2.48 mm^−^^1^
β = 93.481 (2)º	*T* = 298 (2) K
*V* = 1268.5 (4) Å^3^	Block, green
*Z* = 2	0.65 × 0.10 × 0.07 mm

### Data collection

**Table:**

Bruker SMART CCD area-detector diffractometer	4255 independent reflections
Radiation source: fine-focus sealed tube	2269 reflections with *I* > 2σ(*I*)
Monochromator: graphite	*R*_int_ = 0.053
*T* = 298(2) K	θ_max_ = 25.0º
φ and ω scans	θ_min_ = 1.9º
Absorption correction: multi-scan(SADABS; Sheldrick, 1996)	*h* = −12→10
*T*_min_ = 0.296, *T*_max_ = 0.846	*k* = −7→7
6692 measured reflections	*l* = −23→21

### Refinement

**Table:**

Refinement on *F*^2^	Hydrogen site location: inferred from neighbouring sites
Least-squares matrix: full	H-atom parameters constrained
*R*[*F*^2^ > 2σ(*F*^2^)] = 0.054	*w* = 1/[σ^2^(*F*_o_^2^) + (0.0003*P*)^2^] where *P* = (*F*_o_^2^ + 2*F*_c_^2^)/3
*wR*(*F*^2^) = 0.074	(Δ/σ)_max_ = 0.001
*S* = 0.96	Δρ_max_ = 0.52 e Å^−^^3^
4255 reflections	Δρ_min_ = −0.26 e Å^−^^3^
318 parameters	Extinction correction: none
1 restraint	Absolute structure: Flack (1983), 1785 Friedel pairs
Primary atom site location: structure-invariant direct methods	Flack parameter: 0.054 (14)
Secondary atom site location: difference Fourier map	

### Special details

**Table:**

Geometry. All e.s.d.'s (except the e.s.d. in the dihedral angle between two l.s. planes) are estimated using the full covariance matrix. The cell e.s.d.'s are taken into account individually in the estimation of e.s.d.'s in distances, angles and torsion angles; correlations between e.s.d.'s in cell parameters are only used when they are defined by crystal symmetry. An approximate (isotropic) treatment of cell e.s.d.'s is used for estimating e.s.d.'s involving l.s. planes.
Refinement. Refinement of *F*^2^ against ALL reflections. The weighted *R*-factor *wR* and goodness of fit *S* are based on *F*^2^, conventional *R*-factors *R* are based on *F*, with *F* set to zero for negative *F*^2^. The threshold expression of *F*^2^ > σ(*F*^2^) is used only for calculating *R*-factors(gt) *etc*. and is not relevant to the choice of reflections for refinement. *R*-factors based on *F*^2^ are statistically about twice as large as those based on *F*, and *R*- factors based on ALL data will be even larger.

### Fractional atomic coordinates and isotropic or equivalent isotropic displacement parameters (Å^2^)

**Table:**

	*x*	*y*	*z*	*U*_iso_*/*U*_eq_	
Cu1	0.13990 (7)	0.36558 (17)	0.76571 (4)	0.0503 (3)	
Br1	−0.25268 (7)	0.10824 (17)	0.46920 (4)	0.0793 (3)	
N1	0.2064 (5)	0.1850 (11)	0.6962 (3)	0.0440 (18)	
N2	0.0762 (6)	0.5406 (11)	0.8407 (3)	0.052 (2)	
N3	−0.0327 (5)	0.1749 (11)	0.7873 (3)	0.0451 (18)	
O1	0.2695 (4)	0.2179 (10)	0.8245 (2)	0.0594 (17)	
O2	0.4200 (4)	−0.0388 (10)	0.8229 (2)	0.077 (2)	
O3	0.0499 (4)	0.5429 (9)	0.6982 (2)	0.0540 (17)	
O4	0.4374 (5)	0.7917 (11)	0.9519 (3)	0.115 (3)	
H26	0.5106	0.7840	0.9717	0.172*	
H27	0.4480	0.7869	0.9097	0.172*	
O5	0.3458 (5)	0.3755 (14)	0.9595 (3)	0.124 (2)	
H28	0.3213	0.3217	0.9213	0.186*	
H29	0.3693	0.5068	0.9516	0.186*	
C1	0.3385 (7)	0.0792 (17)	0.7944 (4)	0.057 (2)	
C2	0.3235 (5)	0.0722 (14)	0.7175 (3)	0.050 (2)	
H2	0.3166	−0.0826	0.7032	0.060*	
C3	0.4394 (6)	0.1737 (12)	0.6871 (3)	0.056 (2)	
H3	0.5136	0.1047	0.7101	0.067*	
C4	0.4473 (6)	0.4206 (12)	0.7027 (4)	0.068 (3)	
H4A	0.3766	0.4937	0.6787	0.082*	
H4B	0.4380	0.4410	0.7508	0.082*	
C5	0.5670 (7)	0.5334 (16)	0.6844 (5)	0.130 (5)	
H5A	0.6384	0.4473	0.7009	0.195*	
H5B	0.5715	0.6776	0.7046	0.195*	
H5C	0.5674	0.5471	0.6361	0.195*	
C6	0.4453 (6)	0.1239 (17)	0.6124 (3)	0.080 (3)	
H6A	0.3874	0.2180	0.5868	0.121*	
H6B	0.4229	−0.0278	0.6041	0.121*	
H6C	0.5294	0.1498	0.5989	0.121*	
C7	0.1408 (6)	0.1318 (15)	0.6429 (3)	0.050 (2)	
H7	0.1678	0.0130	0.6178	0.060*	
C8	0.0288 (7)	0.2422 (14)	0.6194 (3)	0.043 (2)	
C9	−0.0084 (7)	0.4464 (14)	0.6466 (4)	0.043 (2)	
C10	−0.1150 (6)	0.5530 (14)	0.6133 (3)	0.055 (3)	
H10	−0.1375	0.6935	0.6273	0.066*	
C11	−0.1843 (7)	0.4556 (15)	0.5618 (4)	0.054 (2)	
H11	−0.2544	0.5280	0.5418	0.065*	
C12	−0.1509 (7)	0.2463 (16)	0.5385 (4)	0.049 (3)	
C13	−0.0444 (6)	0.1447 (15)	0.5654 (3)	0.049 (2)	
H13	−0.0199	0.0097	0.5480	0.059*	
C14	0.1304 (8)	0.7140 (15)	0.8709 (4)	0.065 (3)	
H14	0.2111	0.7518	0.8592	0.078*	
C15	0.0755 (11)	0.843 (2)	0.9188 (4)	0.090 (3)	
H15	0.1181	0.9647	0.9378	0.108*	
C16	−0.0422 (10)	0.7901 (18)	0.9377 (4)	0.084 (4)	
H16	−0.0805	0.8744	0.9700	0.101*	
C17	−0.1043 (9)	0.610 (2)	0.9084 (4)	0.069 (3)	
C18	−0.0414 (8)	0.4876 (14)	0.8610 (4)	0.048 (2)	
C19	−0.1002 (7)	0.2918 (15)	0.8301 (4)	0.050 (2)	
C20	−0.2215 (8)	0.2353 (18)	0.8491 (4)	0.062 (3)	
C21	−0.2734 (8)	0.042 (2)	0.8195 (5)	0.083 (4)	
H21	−0.3541	−0.0035	0.8289	0.100*	
C22	−0.2037 (8)	−0.0789 (18)	0.7768 (4)	0.082 (4)	
H22	−0.2362	−0.2084	0.7573	0.098*	
C23	−0.0860 (8)	−0.0077 (15)	0.7629 (4)	0.063 (3)	
H23	−0.0402	−0.0941	0.7343	0.075*	
C24	−0.2293 (10)	0.536 (2)	0.9246 (5)	0.090 (4)	
H24	−0.2725	0.6154	0.9562	0.108*	
C25	−0.2840 (9)	0.359 (3)	0.8958 (5)	0.094 (4)	
H25	−0.3645	0.3182	0.9069	0.113*	

### Atomic displacement parameters (Å^2^)

**Table:**

	*U*^11^	*U*^22^	*U*^33^	*U*^12^	*U*^13^	*U*^23^
Cu1	0.0480 (5)	0.0569 (7)	0.0457 (6)	−0.0033 (6)	0.0014 (4)	−0.0045 (6)
Br1	0.0785 (6)	0.0890 (9)	0.0672 (6)	−0.0008 (6)	−0.0220 (5)	−0.0151 (7)
N1	0.034 (4)	0.058 (5)	0.040 (4)	−0.009 (3)	0.003 (3)	0.012 (3)
N2	0.056 (5)	0.051 (6)	0.047 (4)	−0.002 (4)	−0.006 (3)	−0.006 (4)
N3	0.053 (4)	0.044 (5)	0.038 (4)	0.008 (4)	0.003 (3)	0.001 (3)
O1	0.053 (3)	0.082 (5)	0.044 (3)	0.005 (3)	0.004 (3)	0.000 (3)
O2	0.058 (3)	0.112 (6)	0.061 (4)	0.019 (3)	0.002 (3)	0.028 (4)
O3	0.062 (3)	0.057 (5)	0.041 (3)	0.000 (3)	−0.010 (2)	−0.010 (3)
O4	0.124 (5)	0.134 (8)	0.083 (5)	−0.028 (5)	−0.019 (4)	0.026 (5)
O5	0.188 (6)	0.110 (6)	0.070 (4)	−0.040 (6)	−0.032 (4)	0.021 (5)
C1	0.050 (6)	0.064 (7)	0.056 (6)	−0.006 (5)	−0.001 (4)	0.013 (6)
C2	0.038 (4)	0.050 (6)	0.061 (5)	−0.006 (4)	−0.005 (4)	0.005 (5)
C3	0.052 (5)	0.063 (8)	0.053 (5)	0.011 (5)	0.007 (4)	0.001 (5)
C4	0.053 (5)	0.064 (9)	0.088 (7)	−0.019 (5)	0.004 (4)	0.011 (6)
C5	0.089 (7)	0.105 (12)	0.195 (12)	−0.034 (8)	0.005 (7)	0.011 (9)
C6	0.077 (5)	0.101 (9)	0.064 (6)	−0.001 (7)	0.003 (4)	−0.004 (7)
C7	0.050 (5)	0.056 (6)	0.046 (5)	0.000 (5)	0.006 (4)	−0.005 (5)
C8	0.046 (5)	0.055 (7)	0.027 (5)	0.000 (4)	0.005 (4)	0.000 (4)
C9	0.052 (5)	0.045 (7)	0.033 (5)	0.006 (4)	0.008 (4)	−0.002 (4)
C10	0.076 (6)	0.047 (7)	0.042 (5)	0.016 (5)	0.000 (4)	−0.009 (5)
C11	0.063 (6)	0.049 (7)	0.047 (6)	0.018 (5)	−0.009 (4)	0.000 (5)
C12	0.044 (5)	0.069 (8)	0.035 (5)	0.000 (5)	0.001 (4)	0.004 (5)
C13	0.065 (5)	0.043 (6)	0.040 (5)	0.001 (5)	0.008 (4)	−0.003 (5)
C14	0.084 (7)	0.051 (7)	0.057 (6)	−0.004 (6)	−0.011 (5)	0.006 (5)
C15	0.177 (10)	0.058 (7)	0.033 (5)	0.006 (10)	−0.008 (6)	−0.009 (6)
C16	0.132 (9)	0.074 (10)	0.046 (6)	0.041 (8)	0.014 (6)	−0.004 (6)
C17	0.095 (7)	0.079 (8)	0.032 (5)	0.018 (8)	0.000 (5)	−0.005 (6)
C18	0.059 (6)	0.056 (6)	0.030 (5)	0.014 (5)	−0.002 (4)	0.002 (4)
C19	0.047 (5)	0.062 (8)	0.040 (5)	−0.004 (5)	−0.004 (4)	0.017 (5)
C20	0.052 (6)	0.079 (8)	0.055 (7)	−0.003 (6)	0.001 (5)	0.014 (6)
C21	0.048 (6)	0.120 (13)	0.081 (8)	−0.009 (7)	−0.010 (5)	0.041 (8)
C22	0.060 (6)	0.105 (11)	0.076 (7)	−0.040 (7)	−0.030 (5)	0.024 (7)
C23	0.073 (7)	0.046 (7)	0.067 (6)	−0.015 (5)	−0.018 (5)	−0.007 (5)
C24	0.089 (9)	0.131 (14)	0.052 (7)	0.048 (8)	0.030 (6)	0.027 (7)
C25	0.070 (7)	0.143 (12)	0.072 (8)	0.027 (10)	0.020 (6)	0.026 (9)

### Geometric parameters (Å, °)

**Table:**

Cu1—O3	1.922 (5)	C6—H6C	0.9600
Cu1—N1	1.924 (6)	C7—C8	1.417 (9)
Cu1—O1	1.963 (5)	C7—H7	0.9300
Cu1—N2	1.975 (6)	C8—C13	1.411 (9)
Cu1—N3	2.229 (6)	C8—C9	1.413 (9)
Br1—C12	1.888 (8)	C9—C10	1.429 (9)
N1—C7	1.269 (7)	C10—C11	1.355 (9)
N1—C2	1.458 (7)	C10—H10	0.9300
N2—C14	1.322 (9)	C11—C12	1.401 (10)
N2—C18	1.373 (8)	C11—H11	0.9300
N3—C23	1.320 (9)	C12—C13	1.367 (9)
N3—C19	1.343 (9)	C13—H13	0.9300
O1—C1	1.284 (9)	C14—C15	1.385 (11)
O2—C1	1.232 (8)	C14—H14	0.9300
O3—C9	1.300 (8)	C15—C16	1.365 (11)
O4—H26	0.8500	C15—H15	0.9300
O4—H27	0.8500	C16—C17	1.384 (13)
O5—H28	0.8501	C16—H16	0.9300
O5—H29	0.8500	C17—C18	1.396 (11)
C1—C2	1.520 (6)	C17—C24	1.454 (12)
C2—C3	1.531 (6)	C18—C19	1.456 (10)
C2—H2	0.9800	C19—C20	1.405 (10)
C3—C6	1.512 (6)	C20—C25	1.389 (12)
C3—C4	1.528 (6)	C20—C21	1.406 (12)
C3—H3	0.9800	C21—C22	1.368 (11)
C4—C5	1.506 (6)	C21—H21	0.9300
C4—H4A	0.9700	C22—C23	1.365 (9)
C4—H4B	0.9700	C22—H22	0.9300
C5—H5A	0.9600	C23—H23	0.9300
C5—H5B	0.9600	C24—C25	1.330 (14)
C5—H5C	0.9600	C24—H24	0.9300
C6—H6A	0.9600	C25—H25	0.9300
C6—H6B	0.9600		
			
O3—Cu1—N1	90.5 (2)	N1—C7—H7	118.0
O3—Cu1—O1	165.3 (2)	C8—C7—H7	118.0
N1—Cu1—O1	83.7 (2)	C13—C8—C9	120.0 (7)
O3—Cu1—N2	92.5 (2)	C13—C8—C7	117.6 (8)
N1—Cu1—N2	177.0 (3)	C9—C8—C7	122.4 (7)
O1—Cu1—N2	93.4 (2)	O3—C9—C8	124.1 (7)
O3—Cu1—N3	92.42 (19)	O3—C9—C10	119.0 (8)
N1—Cu1—N3	100.6 (2)	C8—C9—C10	116.9 (7)
O1—Cu1—N3	101.9 (2)	C11—C10—C9	121.9 (8)
N2—Cu1—N3	79.1 (3)	C11—C10—H10	119.0
C7—N1—C2	122.5 (7)	C9—C10—H10	119.0
C7—N1—Cu1	122.0 (5)	C10—C11—C12	120.2 (8)
C2—N1—Cu1	113.8 (4)	C10—C11—H11	119.9
C14—N2—C18	115.6 (7)	C12—C11—H11	119.9
C14—N2—Cu1	127.3 (7)	C13—C12—C11	120.0 (8)
C18—N2—Cu1	116.9 (6)	C13—C12—Br1	120.5 (7)
C23—N3—C19	115.9 (7)	C11—C12—Br1	119.5 (7)
C23—N3—Cu1	134.7 (6)	C12—C13—C8	120.8 (8)
C19—N3—Cu1	109.0 (5)	C12—C13—H13	119.6
C1—O1—Cu1	115.0 (5)	C8—C13—H13	119.6
C9—O3—Cu1	119.2 (5)	N2—C14—C15	124.6 (9)
H26—O4—H27	106.2	N2—C14—H14	117.7
H28—O5—H29	105.7	C15—C14—H14	117.7
O2—C1—O1	124.8 (7)	C16—C15—C14	119.0 (11)
O2—C1—C2	118.0 (8)	C16—C15—H15	120.5
O1—C1—C2	117.0 (7)	C14—C15—H15	120.5
N1—C2—C1	108.2 (6)	C15—C16—C17	119.6 (10)
N1—C2—C3	112.9 (6)	C15—C16—H16	120.2
C1—C2—C3	110.1 (6)	C17—C16—H16	120.2
N1—C2—H2	108.5	C16—C17—C18	117.6 (10)
C1—C2—H2	108.5	C16—C17—C24	124.6 (11)
C3—C2—H2	108.5	C18—C17—C24	117.8 (11)
C6—C3—C4	112.8 (7)	N2—C18—C17	123.7 (9)
C6—C3—C2	112.7 (6)	N2—C18—C19	116.3 (8)
C4—C3—C2	110.4 (6)	C17—C18—C19	120.1 (9)
C6—C3—H3	106.8	N3—C19—C20	125.0 (9)
C4—C3—H3	106.8	N3—C19—C18	117.4 (7)
C2—C3—H3	106.8	C20—C19—C18	117.6 (9)
C5—C4—C3	115.6 (7)	C25—C20—C19	121.9 (10)
C5—C4—H4A	108.4	C25—C20—C21	122.3 (11)
C3—C4—H4A	108.4	C19—C20—C21	115.7 (9)
C5—C4—H4B	108.4	C22—C21—C20	119.2 (10)
C3—C4—H4B	108.4	C22—C21—H21	120.4
H4A—C4—H4B	107.4	C20—C21—H21	120.4
C4—C5—H5A	109.5	C23—C22—C21	119.4 (10)
C4—C5—H5B	109.5	C23—C22—H22	120.3
H5A—C5—H5B	109.5	C21—C22—H22	120.3
C4—C5—H5C	109.5	N3—C23—C22	124.7 (9)
H5A—C5—H5C	109.5	N3—C23—H23	117.7
H5B—C5—H5C	109.5	C22—C23—H23	117.7
C3—C6—H6A	109.5	C25—C24—C17	122.2 (11)
C3—C6—H6B	109.5	C25—C24—H24	118.9
H6A—C6—H6B	109.5	C17—C24—H24	118.9
C3—C6—H6C	109.5	C24—C25—C20	120.4 (11)
H6A—C6—H6C	109.5	C24—C25—H25	119.8
H6B—C6—H6C	109.5	C20—C25—H25	119.8
N1—C7—C8	124.1 (8)		
			
O3—Cu1—N1—C7	−38.3 (6)	Cu1—O3—C9—C8	−28.3 (9)
O1—Cu1—N1—C7	155.2 (6)	Cu1—O3—C9—C10	153.4 (5)
N2—Cu1—N1—C7	139 (5)	C13—C8—C9—O3	176.7 (7)
N3—Cu1—N1—C7	54.2 (7)	C7—C8—C9—O3	−5.4 (11)
O3—Cu1—N1—C2	156.3 (5)	C13—C8—C9—C10	−5.0 (10)
O1—Cu1—N1—C2	−10.2 (5)	C7—C8—C9—C10	172.9 (7)
N2—Cu1—N1—C2	−26 (5)	O3—C9—C10—C11	−176.1 (7)
N3—Cu1—N1—C2	−111.1 (5)	C8—C9—C10—C11	5.6 (11)
O3—Cu1—N2—C14	−92.1 (6)	C9—C10—C11—C12	−1.4 (12)
N1—Cu1—N2—C14	91 (5)	C10—C11—C12—C13	−3.4 (11)
O1—Cu1—N2—C14	74.5 (6)	C10—C11—C12—Br1	177.4 (6)
N3—Cu1—N2—C14	175.9 (6)	C11—C12—C13—C8	3.8 (11)
O3—Cu1—N2—C18	83.0 (5)	Br1—C12—C13—C8	−177.0 (5)
N1—Cu1—N2—C18	−94 (5)	C9—C8—C13—C12	0.5 (10)
O1—Cu1—N2—C18	−110.5 (5)	C7—C8—C13—C12	−177.5 (6)
N3—Cu1—N2—C18	−9.0 (5)	C18—N2—C14—C15	−1.7 (11)
O3—Cu1—N3—C23	90.0 (7)	Cu1—N2—C14—C15	173.4 (7)
N1—Cu1—N3—C23	−1.0 (7)	N2—C14—C15—C16	0.9 (14)
O1—Cu1—N3—C23	−86.7 (7)	C14—C15—C16—C17	−0.4 (14)
N2—Cu1—N3—C23	−177.9 (7)	C15—C16—C17—C18	0.9 (14)
O3—Cu1—N3—C19	−82.0 (5)	C15—C16—C17—C24	179.7 (9)
N1—Cu1—N3—C19	−173.0 (5)	C14—N2—C18—C17	2.3 (11)
O1—Cu1—N3—C19	101.3 (5)	Cu1—N2—C18—C17	−173.4 (6)
N2—Cu1—N3—C19	10.1 (5)	C14—N2—C18—C19	−177.6 (6)
O3—Cu1—O1—C1	−66.4 (12)	Cu1—N2—C18—C19	6.7 (8)
N1—Cu1—O1—C1	1.0 (6)	C16—C17—C18—N2	−1.9 (13)
N2—Cu1—O1—C1	−179.9 (6)	C24—C17—C18—N2	179.3 (8)
N3—Cu1—O1—C1	100.6 (6)	C16—C17—C18—C19	178.0 (7)
N1—Cu1—O3—C9	42.5 (5)	C24—C17—C18—C19	−0.8 (12)
O1—Cu1—O3—C9	109.1 (10)	C23—N3—C19—C20	−1.1 (11)
N2—Cu1—O3—C9	−137.3 (5)	Cu1—N3—C19—C20	172.5 (6)
N3—Cu1—O3—C9	−58.1 (5)	C23—N3—C19—C18	176.6 (6)
Cu1—O1—C1—O2	−175.7 (7)	Cu1—N3—C19—C18	−9.7 (7)
Cu1—O1—C1—C2	8.3 (9)	N2—C18—C19—N3	3.1 (9)
C7—N1—C2—C1	−149.3 (7)	C17—C18—C19—N3	−176.8 (7)
Cu1—N1—C2—C1	16.0 (7)	N2—C18—C19—C20	−179.0 (7)
C7—N1—C2—C3	88.5 (8)	C17—C18—C19—C20	1.1 (11)
Cu1—N1—C2—C3	−106.2 (5)	N3—C19—C20—C25	177.4 (8)
O2—C1—C2—N1	167.9 (7)	C18—C19—C20—C25	−0.3 (12)
O1—C1—C2—N1	−15.8 (10)	N3—C19—C20—C21	−0.6 (11)
O2—C1—C2—C3	−68.2 (10)	C18—C19—C20—C21	−178.3 (7)
O1—C1—C2—C3	108.0 (8)	C25—C20—C21—C22	−176.4 (9)
N1—C2—C3—C6	−72.6 (8)	C19—C20—C21—C22	1.6 (12)
C1—C2—C3—C6	166.3 (8)	C20—C21—C22—C23	−0.9 (14)
N1—C2—C3—C4	54.6 (8)	C19—N3—C23—C22	2.0 (11)
C1—C2—C3—C4	−66.5 (9)	Cu1—N3—C23—C22	−169.6 (6)
C6—C3—C4—C5	−61.5 (9)	C21—C22—C23—N3	−1.0 (13)
C2—C3—C4—C5	171.4 (6)	C16—C17—C24—C25	−178.9 (11)
C2—N1—C7—C8	−177.8 (6)	C18—C17—C24—C25	−0.1 (15)
Cu1—N1—C7—C8	18.1 (10)	C17—C24—C25—C20	0.9 (18)
N1—C7—C8—C13	−170.6 (7)	C19—C20—C25—C24	−0.6 (17)
N1—C7—C8—C9	11.5 (11)	C21—C20—C25—C24	177.2 (10)

### Hydrogen-bond geometry (Å, °)

**Table:**

*D*—H···*A*	*D*—H	H···*A*	*D*···*A*	*D*—H···*A*
O5—H28···O1	0.85	2.06	2.904 (8)	174
O5—H29···O4	0.85	1.87	2.708 (10)	168
O4—H26···O5^i^	0.85	2.06	2.853 (8)	156
O4—H27···O2^ii^	0.85	2.02	2.746 (7)	143

Symmetry codes:  (i) −*x*+1, *y*+1/2, −*z*+2; (ii) *x*, *y*+1, *z*.
